# Unveiling the Potential of B_3_O_3_ Nanoflake as Effective Transporter for the Antiviral Drug Favipiravir: Density Functional Theory Analysis

**DOI:** 10.3390/molecules28248092

**Published:** 2023-12-14

**Authors:** Muhammad Nauman Zahid, Naveen Kosar, Hasnain Sajid, Khalid Elfaki Ibrahim, Mansour K. Gatasheh, Tariq Mahmood

**Affiliations:** 1 Department of Biology, College of Science, University of Bahrain, Sakhir P.O. Box 32038, Bahrain; nzahid@uob.edu.bh; 2 Department of Chemistry, University of Management and Technology (UMT), C-11, Johar Town Lahore, Lahore 54770, Pakistan; naveenflavia@gmail.com; 3 School of Science and Technology, Nottingham Trent University, Clifton Lane, Nottingham NG11 8NS, UK; hasnainmsajid@yahoo.com; 4 Department of Zoology, College of Science, King Saud University, P.O. Box 2455, Riyadh 11451, Saudi Arabia; kibrahim@ksu.edu.sa; 5 Department of Biochemistry, College of Science, King Saud University, P.O. Box 2455, Riyadh 11451, Saudi Arabia; mgatasheh@ksu.edu.sa; 6 Department of Chemistry, COMSATS University, Abbottabad Campus, Abbottabad 22060, Pakistan; 7 Department of Chemistry, College of Science, University of Bahrain, Sakhir P.O. Box 32038, Bahrain

**Keywords:** B_3_O_3_, Favipiravir, DFT, Drug delivery, QTAIM

## Abstract

In this study, for the first time, boron oxide nanoflake is analyzed as drug carrier for favipiravir using computational studies. The thermodynamic stability of the boron oxide and favipiravir justifies the strong interaction between both species. Four orientations are investigated for the interaction between the favipiravir and the B_3_O_3_ nanoflake. The E_int_ of the most stable orientation is −26.98 kcal/mol, whereas the counterpoise-corrected energy is −22.59 kcal/mol. Noncovalent interaction index (NCI) and quantum theory of atoms in molecules (QTAIM) analyses are performed to obtain insights about the behavior and the types of interactions that occur between B_3_O_3_ nanoflake and favipiravir. The results indicate the presence of hydrogen bonding between the hydrogen in the favipiravir and the oxygen in the B_3_O_3_ nanoflake in the most stable complex (FAV@B_3_O_3_-C1). The electronic properties are investigated through frontier molecular orbital analysis, dipole moments and chemical reactivity descriptors. These parameters showed the significant activity of B_3_O_3_ for favipiravir. NBO charge analysis transfer illustrated the charge transfer between the two species, and UV-VIS analysis confirmed the electronic excitation. Our work suggested a suitable drug carrier system for the antiviral drug favipiravir, which can be considered by the experimentalist for better drug delivery systems.

## 1. Introduction

Favipiravir (FAV) is an antiviral drug that has shown potential in the treatment of several RNA viruses, including influenza, Ebola virus, and, most recently, SARS-CoV-2, the virus responsible for the COVID-19 pandemic [1]. Favipiravir, also known as T-705, was initially developed as an influenza drug and works by selectively inhibiting the RNA-dependent RNA polymerase (RdRp) of the virus, there by preventing viral replication [2]. The chemical name of favipiravir is 6-fluoro-3-hydroxy-2-pyrazine carboxamide, and its molecular formula is C_5_H_4_FN_3_O. The structure of favipiravir consists of a pyrazine ring linked to a carboxamide group, with a fluorine atom and a hydroxyl group attached at positions 6 and 3 [3].

In a study conducted by Furuta et al. (2013), favipiravir demonstrated potent antiviral activity against the influenza virus in vitro and in animal models [2]. Additionally, in a clinical trial conducted in Japan during the 2014 Ebola outbreak, favipiravir showed efficacy in reducing mortality rates [4]. More recently, several studies have evaluated the efficacy of favipiravir as a treatment for COVID-19. In a randomized clinical trial conducted in China, patients treated with favipiravir had a shorter time for fever resolution and improved radiological findings compared to the control group [5]. In another study conducted in India, favipiravir was found to significantly improve clinical recovery in COVID-19 patients compared to standard care [6]. Overall, favipiravir has shown promise as a potential treatment for several RNA viruses, including SARS-CoV-2.

Drug delivery systems (DDS) refer to the technologies used to deliver therapeutic agents to their intended targets in the body. The development of DDS has revolutionized the field of medicine by enabling targeted and controlled drug release, improving therapeutic efficacy, and minimizing side effects [7,8]. One promising area of research in DDS is the use of nanomaterials for drug delivery, which offers advantages such as high drug loading capacity, prolonged circulation time, and enhanced bioavailability [9,10]. 

Various nanostructures, including nanosheets, nanocages, and nanoparticles, have been successfully employed for drug delivery systems, as reported in the literature [11,12,13,14,15,16,17,18]. Among these, carbon-based nanomaterials have become increasingly popular due to their high efficiency [19,20,21,22]. For instance, graphdiyne has been used to effectively deliver sorafenib and regorafenib [23]. While graphene sheets have also been reported for drug delivery systems [24,25], their limited chemical mobility restricts their use for many drugs [26,27]. Recent studies indicate that boron oxide nanosheets (B_3_O_3_) offer several advantages over graphene nanosheets. B_3_O_3_ has a reactive hollow cavity in comparison to graphene, which makes it a more attractive option [28].

Boron oxide nanosheets (B_3_O_3_) are a type of two-dimensional nanomaterial with a hexagonal lattice structure. B_3_O_3_ nanosheets have been investigated for various applications, including catalysis, electronic devices, and biomedical applications such as drug delivery [29,30,31,32]. Experimental and theoretical studies have confirmed that B_3_O_3_ nanosheets possess a hexagonal planar structure, with strong covalent bonds between the boron atoms. The six-fold symmetry of this structure has been reported in both experimental [33] and theoretical studies [34]. The formation of B_3_O_3_ nanosheets occurs through the condensation of three tetrahydroxydiboron molecules [35], which connect six B_3_O_3_ hexagons to create a porous structure with a surface area of 2.32 Å. This porous structure is ideal for attracting analytes. In 2018, Lin et al. theoretically designed a porous B_3_O_3_ nanosheet [36] with a flat surface with identical pores, as reported in the literature. B_3_O_3_ nanosheets have shown promise as a DDS due to their biocompatibility, low toxicity, and ability to encapsulate drugs and release them in a controlled manner [35,37,38].

B_3_O_3_ nanosheets have shown promise for delivering anticancer agents. For example, the nanosheets were functionalized with a targeting agent and demonstrated enhanced accumulation in cancer cells, leading to increased therapeutic efficacy [39]. B_3_O_3_ monolayers have been investigated as potential carriers for a flutamide-based anticancer drug delivery system [36]. Similarly, magnetic boron nitride nanosheets have been utilized as pH-responsive smart nanocarriers for the delivery of doxorubicin in the treatment of liver cancer [40]. 

Our study will employ density functional theory (DFT) calculations, powerful tools for rationalizing experimentally observed phenomena and predicting the behavior, properties, and applications of various systems [41,42]. Despite their potential, there are no reports in the literature exploring the use of B_3_O_3_ nanoflake as antiviral drug carrier for favipiravir. Therefore, we aim to propose B_3_O_3_ as a drug carrier for this antiviral drug. We hypothesize that B_3_O_3_ has the potential to serve as a drug carrier for favipiravir. Our results support our hypothesis, as we observed excellent interaction energies between favipiravir and B_3_O_3_ nanoflakes.

## 2. Results

### 2.1. Geometric and Energetic Analysis

**Figure 1:**
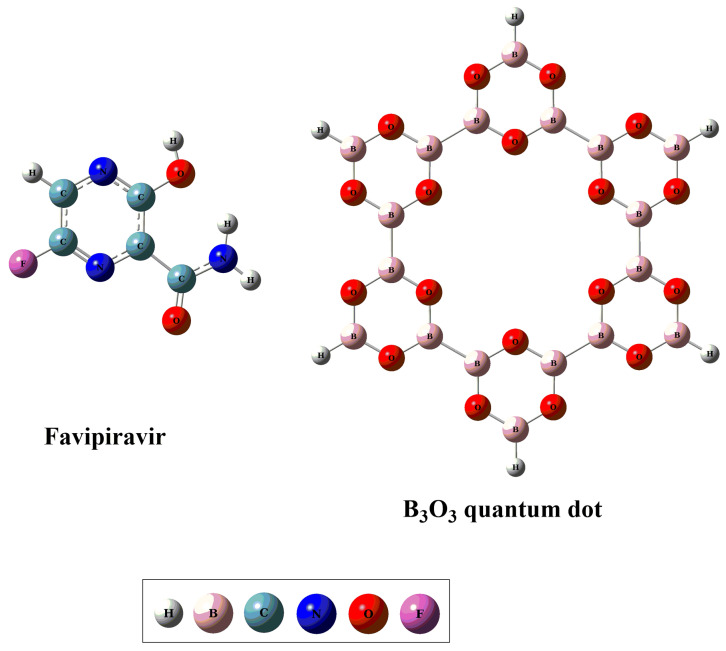
The optimized geometries of bare favipiravir and the B_3_O_3_ nanoflake.

**Figure 2:**
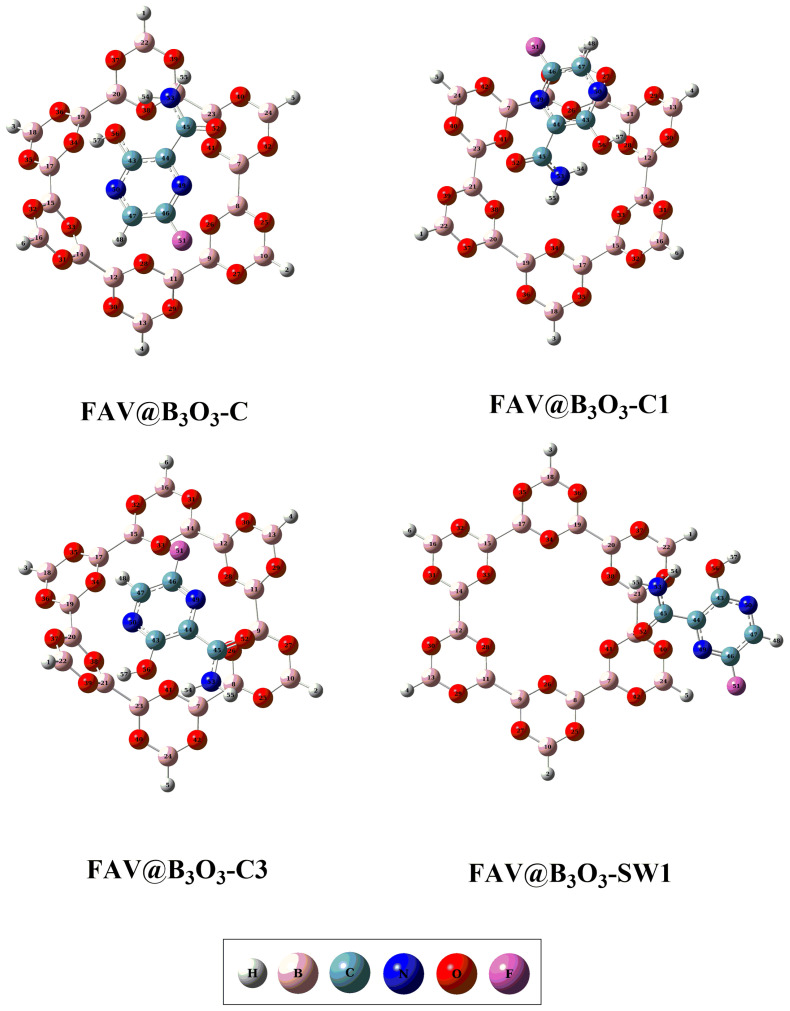
The optimized geometries of FAV@B_3_O_3_ nanoflake complexes such as FAV@B_3_O_3_-C, FAV@B_3_O_3_-C1, FAV@B_3_O_3_-C3 and FAV@B_3_O_3_-SW1.

For exploring the interaction of favipiravir with B_3_O_3_ nanoflake, four different orientations are chosen. These orientations are as follows: (i) favipiravir is adsorbed horizontally on the B_3_O_3_ surface (FAV@ B_3_O_3_-C); (ii) the amino group is directed toward the center of the B_3_O_3_ surface (FAV@ B_3_O_3_-C1); (iii) the aromatic ring containing fluoride is directed towards the center of the B_3_O_3_ surface (FAV@ B_3_O_3_-C3); and (iv) favipiravir is adsorbed on the side of the B_3_O_3_ surface (FAV@ B_3_O_3_-SW1). The optimized energy minima structures of the individual drug, B_3_O_3_ nanoflakes and all complexes are given in Figure 1 and Figure 2. The prominent interacting distances (d_int_), interaction energies (E_int_) and counterpoise-counterpoise energies (Ecp) are summarized in Table 1. Geometric optimization is followed by vibrational analysis, which confirmed that these optimized structures represent the true minima on the potential energy surface. The optimized configurations revealed the highly reactive nature of the porous cavity of B_3_O_3_ and its strong propensity for binding with incoming molecules. The respective observed counterpoise-corrected energies (E_cp_) for the optimized complexes were −20.08, −22.59, −20.70 and −10.66 kcal/mol for the orientations FAV@B_3_O_3_-C, FAV@B_3_O_3_-C1, FAV@ B_3_O_3_-C3 and FAV@ B_3_O_3_-SW1, respectively. The E_cp_ values are comparable to interaction energies (E_int_)—see Table 1. The reason for the stability (as shown by exothermic reactions) of these complexes is the presence of various strong noncovalent interactions between the drug and the surface.

The larger Ecp value (−22.59 kcal mol^−1^) for FAV@B_3_O_3_-C1 indicates that the NH_2_ group of the drug possesses more electropositive protons, which can form strong interactions with the oxygen atoms of B_3_O_3_, resulting in higher interaction energy. The reason for these strong interactions is the presence of oxygen, which has great affinity for the electropositive hydrogen atoms of the amine group. Consequently, these highly electropositive hydrogen atoms exhibit stronger interactions with the electron-rich cavities of the B_3_O_3_ surface through hydrogen bonding. FAV@B_3_O_3_-C1 has two hydrogen bonds, leading to a higher Ecp compared to FAV@B_3_O_3_-C, which has only one hydrogen bond between the hydrogen of the hydroxyl group of favipiravir and the oxygen of the B_3_O_3_ surface. However, in FAV@B_3_O_3_-C3, an additional interaction occurs between the fluoride of favipiravir and the boron atoms of the B_3_O_3_ surface. The additional halogen interaction between fluoride and the surface gives more stability to FAV@B_3_O_3_-C3 compared to FAV@B_3_O_3_-C. Due to the fewer interactions and lower electronegativity on the sides of B_3_O_3_, the Ecp value for FAV@ B_3_O_3_-SW1 is significantly lower than that of the other three complexes (FAV@B_3_O_3_-C, FAV@B_3_O_3_-C1 and FAV@B_3_O_3_-C3). These findings also demonstrate that Ecp increases as the interacting distances (d_int_) decrease. The important interaction distances are given in Table 1, and the interacting distance (d_int_) of the first bond is up to 2 Å for all complexes except for the FAV@B_3_O_3_-SW1 complex. The d_int_ of the second bond is lower for FAV@B_3_O_3_-C1 (2.27 Å), followed by FAV@B_3_O_3_-C3 (2.57 Å) and FAV@B_3_O_3_-C (2.70 Å). The largest d_int_ is seen for FAV@B_3_O_3_-SW1 (3.01 Å). These results align with the existing literature indicating an inverse relationship between thermodynamic stability and distance [43,44].

### 2.2. Noncovalent Interactions (NCI) Analysis

**Figure 3:**
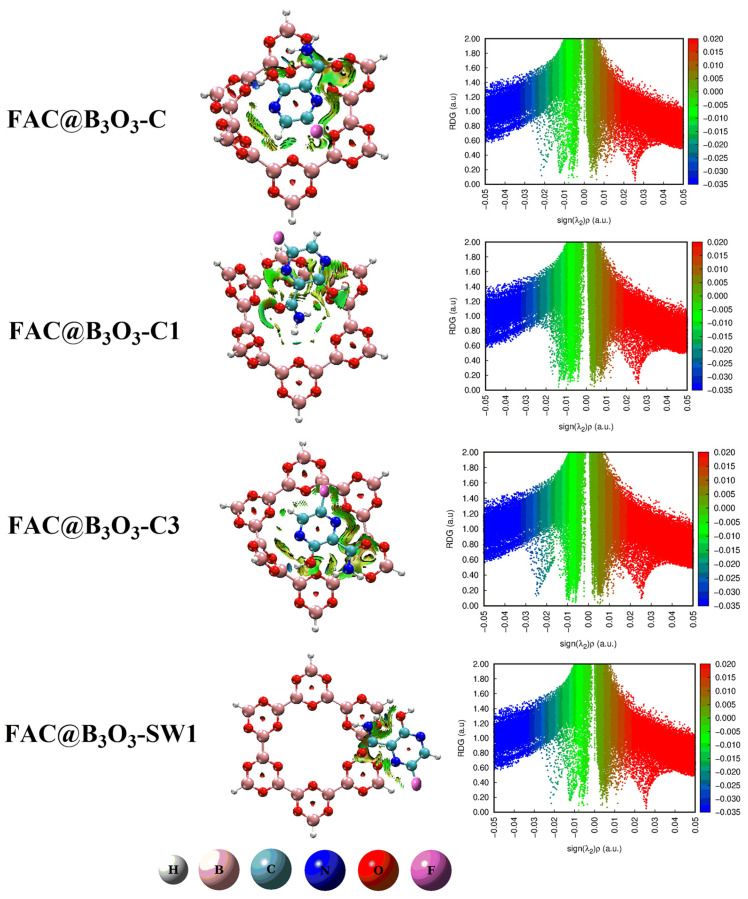
Two-dimensional RDG scattered graphs (right) and 3D isosurfaces (left) of favipiravir@B_3_O_3_ complexes (FAV@B_3_O_3_-C, FAV@B_3_O_3_-C1, FAV@B_3_O_3_-C3, FAV@B_3_O_3_-SW1).

The noncovalent interactions between the surface and analyte (drug) for the deeper visualization are evaluated through NCI analysis. The results of the NCI plots are presented in two forms; 2D RDG scattered graphs and 3D isosurfaces. The 2D RDG scattered graphs and 3D isosurfaces for our designed complexes are given in Figure 3. In this analysis, the nature of the interactions is represented by three colors, namely red, blue and green. The blue, green and red colors represent strong hydrogen bonding interactions, London dispersion interactions and steric repulsion between the analyte and surface, respectively [38,45,46,47].

The scattered graphs of RDG are generated on the basis of the mathematical equation given below.

$$RDG=\frac{1}{{2\left(3\pi^{2}\right)}^{1/3\;}}\frac{\left|\nabla \rho \right|}{\rho^{4/3}}$$

where ∇ρ represents the average reduced density gradient. The *λ*_2_ term in the sign *λ*_2_(ρ) function is obtained using the RDG method as the second largest eigenvalue of the average electron density Hessian matrix computed throughout the dynamical trajectory. It provides information about the different types of weak interactions in a system. The existence of green spikes between −0.020 and 0.001 au in the 2D RDG map evidences the presence of dominant dispersion forces (van der Waals forces) in all complexes. There are some blue spikes in the 2D RDG map depicting hydrogen bonding between the hydrogen of the amino group (in Favipiravir) and the oxygen of B_3_O_3_ in all complexes. The red spikes in the RDG scatter maps show intermolecular steric repulsion in all complexes. These results indicate a stronger influence of van der Waal’s interactions and hydrogen bonding.

Furthermore, the 3D isosurfaces are plotted at an isosurface value of 0.05 au. The strength of nonbonding interactions between the analyte and the surface is estimated based on the thickness of the patches. The stippled patches show weak interactions, but thick patches indicate strong interactions between the analyte (Favipiravir) and the surface (B_3_O_3_). 

All complexes (FAV@B_3_O_3_-C, FAV@B_3_O_3_-C1, FAV@B_3_O_3_-C3, FAV@B_3_O_3_-SW1) show dark green thick patches which depict strong dispersion interactions between favipiravir and B_3_O_3_. However, the thickness of these patches is less in the FAV@B_3_O_3_-C and FAV@B_3_O_3_-SW1 complexes as compared to the FAV@B_3_O_3_-C1 and FAV@B_3_O_3_-C3 complexes. We also noticed small red patches which illustrate steric repulsion between favipiravir and B_3_O_3_. The blue patches indicate the hydrogen bonding between the hydrogen of the amino groups (in favipiravir) and the oxygen of B_3_O_3_. The most pronounced blue patches are seen in the FAV@B_3_O_3_-C1 complex. The outcomes of both the 2D RDG graphs and the 3D isosurfaces illustrate the stability of these complexes. The results of NCI are consistent with the interaction energy results (vide supra).

### 2.3. Quantum Theory of Atoms in Molecules (QTAIM) Analysis

QTAIM analysis is a topological technique used to estimate the nature and strength of interactions between chemical species such as adsorbent (Favipiravir) and surface (B_3_O_3_). The electronic density (ρ), Laplacian electron density (∇2ρ), and the sum of electron densities (H) are important parameters in QTAIM results to differentiate between the covalent and noncovalent interactions. The sum of electron densities (H) at bond critical points (BCPs) is the sum of kinetic and potential energy densities [48,49], which can be calculated using the equation below.

H = G + V

In the above equation, G and V represent the kinetic and potential energy densities. The H value is either zero or less than zero for all types of noncovalent interaction. Meanwhile, an H value greater than zero indicates significant electronic contribution and represents the covalent nature of the interaction. For close shell interactions, the kinetic energy density dominant over potential energy density where H is positive. The total electronic density produces the total electronic energy when integrated over all of the space [48]. A value of electron density (ρ) less than 0.1 au indicates the presence of non-covalent interactions with a positive value of Laplacian electron density (∇2ρ) and sum of electron densities (H).

**Figure 4:**
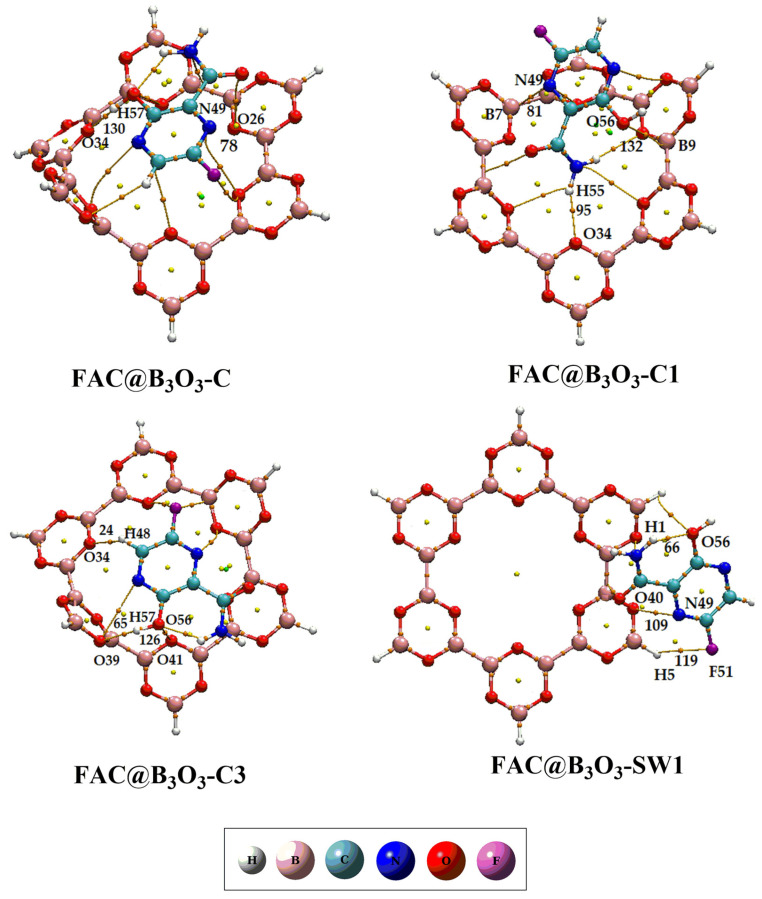
QTAIM analysis of favipiravir@B_3_O_3_ complexes (FAV@B_3_O_3_-C, FAV@B_3_O_3_-C1, FAV@B_3_O_3_-C3, FAV@B_3_O_3_-SW1).

To examine the interactions more deeply and find bond critical points (BCPs) of favipiravir-adsorbed B_3_O_3_ complexes (FAV@B_3_O_3_-C, FAV@B_3_O_3_-C1, FAV@B_3_O_3_-C3, FAV@B_3_O_3_-SW1), the QTAIM analysis is performed, and the results are given in Table 2 and the BCPs are shown in Figure 4.

The average values of ρ in favipiravir-adsorbed B_3_O_3_ complexes such as FAV@B_3_O_3_-C, FAV@B_3_O_3_-C1, FAV@B_3_O_3_-C3, and FAV@B_3_O_3_-SW1 range from 0.80 × 10^−2^ to 0.42 × 10^0^, respectively. The positive values of H in these complexes indicate the presence of non-covalent interactions in all reported complexes. However, the negative values of H in some of the critical points of the complexes also depict the existence of hydrogen bonding, which is comparable to the NCI results (vide supra). 

### 2.4. Electronic Properties

**Figure 5:**
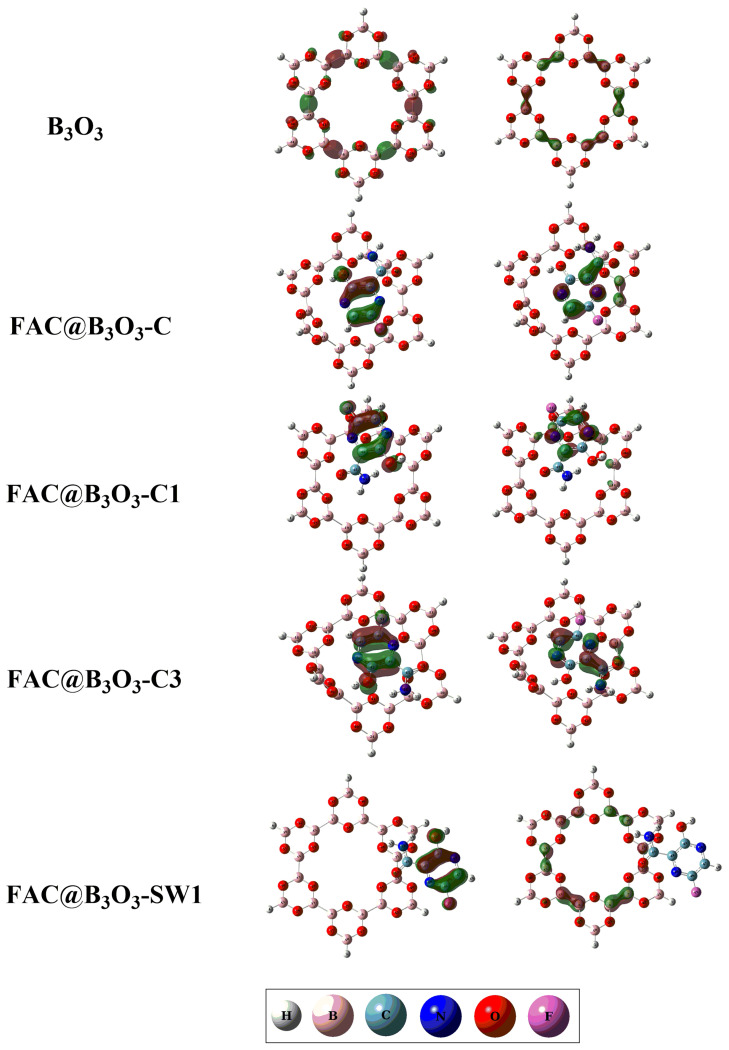
The HOMO and LUMO isosurface of the FAV@B_3_O_3_ complexes (bare B_3_O_3_, favipiravir, FAV@B_3_O_3_-C, FAV@B_3_O_3_-C1, FAV@B_3_O_3_-C3 and FAV@B_3_O_3_-SW1).

The computation of frontier molecular orbitals is carried out to evaluate the electronic properties of drug and B_3_O_3_ nanoflake. According to the literature, the electronic behavior of a surface undergoes changes when they interact with any chemical species [50,51]. Table 3 and Figure 5 exhibit the energies of the HOMO and LUMO orbitals, as well as their corresponding isosurfaces. The energy gap between the HOMO and LUMO of pure B_3_O_3_ is determined to be 9.95 eV. However, upon complexation with the considered drug, the energy gap between HOMO and LUMO orbitals of FAV@B_3_O_3_-C, FAV@B_3_O_3_-C1, FAV@B_3_O_3_-C3 and FAV@B_3_O_3_-SW1 complexes is decreased. Specifically, the energy gap between the MOMO and LUMO orbitals for FAV@B_3_O_3_-C, FAV@B_3_O_3_-C1, FAV@B_3_O_3_-C3 and FAV@B_3_O_3_-SW1 complexes is 8.62, 8.67, 8.61 and 8.70 eV, respectively. The isosurface visualization of the HOMO and LUMO provides insights into the localization of the HOMO on the bonds in drug molecules in all of the doped complexes, while the LUMO is localized on the atoms of the drug as well. The dipole moment is another crucial factor that defines the solubility and polarity of the system [52]. 

For the B_3_O_3_ nanoflakes, the dipole moment is measured to be 0.00 Debye, indicating their non-polar nature. However, the dipole moment in the FAV@B_3_O_3_-C, FAV@B_3_O_3_-C1, FAV@B_3_O_3_-C3 and FAV@B_3_O_3_-SW1 complexes is 5.96 D, 5.46 D, and 6.01 and 6.37 Debye, respectively. The dipole moment values of the reported FAV@B_3_O_3_ complexes demonstrate that the B_3_O_3_ nanoflakes acquire polarity upon interaction with the respective drug. This polarity arises from the Coulombic interactions between the nucleophilic cavities of B_3_O_3_ and the electrophilic hydrogen atoms of the drug in the doped complexes, especially in the FAV@B_3_O_3_-C1, FAV@B_3_O_3_-C3 and FAV@B_3_O_3_-C complexes. The dipole moments of FAV@B_3_O_3_-C1 and FAV@B_3_O_3_-C3 indicate that these complexes are reasonably soluble in an aqueous medium. On the other hand, FAV@B_3_O_3_-SW1 is relatively less soluble due to the absence of Coulombic interactions. Good solubility is typically a desirable property for a drug delivery system, which is evident in the FAV@B_3_O_3_-C1, FAV@B_3_O_3_-C3 and FAV@B_3_O_3_-C complexes.

The reactivity of favipiravir with the B_3_O_3_ quantum dots is evaluated using chemical reactive descriptors such as hardness (*η*), softness (s), chemical potential (*μ*), and electrophilicity index (*ω*) (see Table 3). The results indicate that the chemical potential (*μ*) of the respective drug-doped B_3_O_3_ complexes is higher than that of both the bare B_3_O_3_ nanosheets and the drug molecule. Furthermore, the high softness (s) values and low hardness (*η*) values follow the same trend as the chemical potential, indicating the stability of the doped complexes after complexation when compared to bare B_3_O_3_. Notably, the FAV@B_3_O_3_-C3 complex exhibits the highest softness (0.12 eV) value and the lowest hardness value (4.31 eV), indicating its lower reactivity and higher stability among all of the designed complexes. The FAV@B_3_O_3_-C3 complex also has the highest chemical potential (−5.28 eV) and electrophilicity index (3.23 eV) compared to the other bare and doped complexes. The higher chemical potential indicates greater charge transfer in this FAV@B_3_O_3_-C3 complex and the high electrophilicity index justifies the higher stabilization energy of the doped complex. The FAV@B_3_O_3_-SW1 complex exhibits the lowest softness (0.11 eV) value and the highest hardness value (4.36 eV), indicating lower stability and high reactivity among all designed complexes. On the other hand, the FAV@B_3_O_3_-C1 complex has the lowest chemical potential (−5.26 eV) and electrophilicity index (3.11 eV). These results indicate the lower charge transfer and lower stabilization energy of the FAV@B_3_O_3_-C1 complex. The high electrophilicity, low hardness, high softness, and high chemical potential values collectively suggest that B_3_O_3_ can serve as an effective drug delivery system for favipiravir.

### 2.5. UV-VIS Analysis

**Figure 6:**
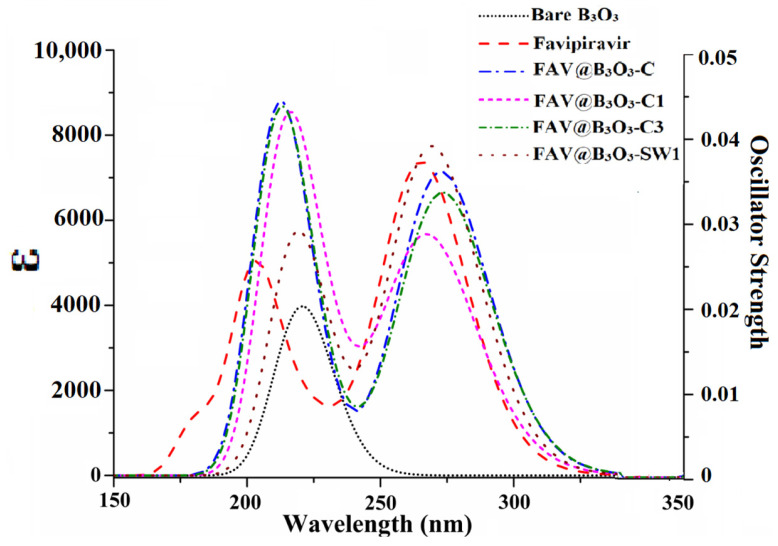
UV-Vis analysis of the FAV@B_3_O_3_ complexes (bare B_3_O_3_, favipiravir, FAV@B_3_O_3_-C, FAV@B_3_O_3_-C1, FAV@B_3_O_3_-C3 and FAV@B_3_O_3_-SW1).

The UV-Vis analysis plays a crucial role in comprehending the behavior of the sensor as an optical sensor. As per the existing literature, a rise in the interaction energy between two chemical species is projected to cause a change in wavelength for an optical sensor [11]. Figure 6 and Table 3 present the UV-Vis spectra and corresponding values for both the bare and complexed B_3_O_3_. The absorbance of bare B_3_O_3_ was observed at 221 nm. However, upon complexation with favipiravir, this absorbance shifts towards higher wavelengths, as observed in our previous report [38]. The *λ*_max_ of B_3_O_3_ experiences a shift to 273 nm, 266 nm, 274 nm and 269 nm for FAV@B_3_O_3_-C, FAV@B_3_O_3_-C1, FAV@B_3_O_3_-C3 and FAV@B_3_O_3_-SW1 complexes, respectively (Figure 6). This red shift is observed for all of the doped complexes. The UV-Vis analysis justifies electronic excitation in doped complexes due to the strong interaction between the drug and the B_3_O_3_ surface. These findings strongly indicate the effectiveness of B_3_O_3_ for optical sensing applications involving the antiviral drug favipiravir.

Internal descriptors including the oscillating strength and excitation energies are calculated to understand the reason for the change in wavelength. The wavelength is directly proportional to the oscillating strength and inversely proportional to the excitation energy. The FAV@B_3_O_3_-C has a maximum wavelength of 273 nm and an oscillating strength of 0.170. The FAV@B_3_O_3_-C1 has a maximum wavelength of 266 nm and an oscillating strength of 0.119. The trend of increasing *f*_o_ is comparable to the increase in wavelength. The excitation energy also decreases for doped complexes compared to bare B_3_O_3_ (5.62 eV). The excitation energy ranges from 4.53 to 4.67 eV. The trend of increasing wavelength is similar to the increase in excitation energy. The lowest excitation energy (4.53 eV) is seen for FAV@B_3_O_3_-C3, which has a maximum wavelength of 274 nm. The lowest excitation energy is 4.66 eV for FAV@B_3_O_3_-C3, which has a maximum wavelength of 266 nm. The excitation energy is the dominating factor responsible for causing changes in the wavelength of the doped complexes (Table 4).

## 3. Materials and Methods

### Computational Methodology

DFT simulations were conducted using Gaussian 09 [53], while the GaussView 5.0 [54] software package was used for visualization. Geometric analysis utilized the ωB97XD functional in conjunction with the 6-31+G(d,p) basis set. The ωB97XD functional is a hybrid and long-range separated functional with additional dispersion correction, and is popular due to its treatment of non-covalent interactions [55,56,57]. This correction factor accounts for weak London dispersion forces and ensures the production of accurate optimization results [58,59]. The interaction (E_int_) and counterpoise-corrected (E_cp_) energies were calculated by using Equations (1) and (2):

(1) $$E_{int}=E_{FAV-B_{3}O_{3}-[E_{FAV}+E_{B_{3}O_{3}}]}$$

(2) $$E_{cp}=E_{FAV-B_{3}O_{3}-\left[E_{FAV}+E_{B_{3}O_{3}}\right]+BSSE}$$

where E_FAV@B3O3_, E_FAV_, and E_B3O3_ represent the energies of the FAV@B_3_O_3_ complexes, favipiravir (FAV), and the B_3_O_3_ surface, respectively. BSSE refers to the basis set superposition error caused by overlapping basis sets, and it was corrected by using the counterpoise method specified in Gaussian 09 [58]. The interaction energies indicate the non-covalent physiosorption of favipiravir onto the surface of the B_3_O_3_ nanoflakes. To evaluate the non-covalent interactive and repulsive forces, non-covalent interaction index (NCI) and quantum theory of atoms in molecules (QTAIM) analyses were performed using Multiwfn 3.8 software [60].

Electronic properties were investigated at the ωB97XD/6-31+G(d,p) level of theory. ωB97XD is a highly reliable functional widely used for investigating the electronic properties of various systems, providing energy gaps comparable to experimental data [61,62,63]. NBO charge transfer calculations were conducted to determine the extent of charge transfer between the interacting moieties (drug and surface). Additionally, electronic descriptors such as the chemical hardness, softness, chemical potential, and electrophilicity index were computed to analyze the reactivity of the systems:

(3) $$\eta =E_{LUMO}-E_{HOMO}/2$$

S = 1/2*η* (4)

(5) $$µ=E_{HOMO}+E_{LUMO}/2$$

*ω* = *μ*^2^/2*η* (6)

## 4. Conclusions

B_3_O_3_ nanoflakes are analyzed as drug carriers for the antiviral drug favipiravir using DFT simulations. The strong interactions between both species (boron oxide and favipiravir) depict their thermodynamic stability. Four orientations are investigated for the interaction between favipiravir and the B_3_O_3_. The E_int_ of the most stable orientation is −26.98 kcal/mol, whereas the counterpoise-corrected energy is −22.59 kcal/mol. The electronic properties are investigated through frontier molecular orbital analysis, dipole moments and chemical reactivity descriptors. These parameters indicate the significant activity of B_3_O_3_ nanoflakes for favipiravir. NBO charge transfer illustrates the charge transfer between the interacting species. Noncovalent interaction index (NCI) and quantum theory of atoms in molecules (QTAIM) analyses are performed to gain insights about the behavior and the types of interactions that occur between B_3_O_3_ quantum dots and favipiravir. The results indicate the presence of hydrogen bonding between the hydrogen of the favipiravir and the oxygen of the B_3_O_3_ quantum dots in the most stable complex (FAV@B_3_O_3_-C1). UV-Vis analysis confirmed the electronic excitation. All of the complexes showed red shift compared to bare B_3_O_3_ quantum dots and favipiravir. Our work provides a suitable drug carrier system for the antiviral drug favipiravir, which can be considered by the experimentalist for better drug delivery systems.

## Figures and Tables

**Table 1:** Interaction energies (E_int_), interacting atoms (A_int_), interacting distances (d_int_), and counterpoise-corrected energies (E_cp_) of reported FAV@B_3_O_3_ complexes.

Complexes	E_int_ (kcal/mol)	A_int_	d_int_ (Å)	E_cp_ (kcal/mol)
FAV@B_3_O_3_-C	−23.84	H57—O34	1.84	−20.08
		H48—O28	2.70	
FAV@B_3_O_3_-C1	−26.98	H55—O34	2.13	−22.59
		H54—O28	2.27	
FAV@B_3_O_3_-C3	−24.47	H57—O39	1.94	−20.70
		H48—O34	2.57	
FAV@B_3_O_3_-SW1	−12.55	O52—B23	2.71	−10.66
		N49—O40	3.01	

**Table 2:** QTAIM parameters including the electronic density (ρ), Laplacian electron density (∇2ρ), kinetic energy density (G), potential energy density (V) and the sum of electron densities (H) at bond critical points (BCPs) in favipiravir-adsorbed B_3_O_3_ complexes (B_3_O_3_-1-FAV-C, B_3_O_3_-1-FAV-C-1, B_3_O_3_-1-FAV-C-3, and B_3_O_3_-1-FAV-C-SW1 in au).

BCPs	Ana-Surface	ρ	∇^2^ρ	G	V	H
FAV@B_3_O_3_-C						
78	N49-O26	0.60 × 10^−2^	0.21 × 10^−1^	0.44 × 10^−2^	−0.36 × 10^−2^	0.79 × 10^−3^
130	H57-O34	0.31 × 10^−1^	0.94 × 10^−1^	0.23 × 10^−1^	−0.23 × 10^−1^	0.92 × 10^−4^
FAV@B_3_O_3_-C1						
81	N49-B7	0.80 × 10^−2^	0.24 × 10^−1^	0.52 × 10^−2^	−0.44 × 10^−2^	0.79 × 10^−3^
95	H55-O34	0.17 × 10^−1^	0.52 × 10^−1^	0.13 × 10^−1^	−0.13 × 10^−1^	−0.26 × 10^−3^
132	O56-B9	0.12 × 10^−1^	0.33 × 10^−1^	0.80 × 10^−2^	−0.77 × 10^−2^	0.22 × 10^−3^
FAV@B_3_O_3_-C3						
24	H48-O34	0.42 × 10^0^	−0.18 × 10^2^	0.68 × 10^−2^	−0.46 × 10^1^	−0.46 × 10^1^
65	H57-O39	0.25 × 10^−1^	0.71 × 10^−1^	0.18 × 10^−1^	−0.19 × 10^−1^	0.49 × 10^3^
126	O56-O41	0.11 × 10^−1^	0.35 × 10^−1^	0.82 × 10^−2^	−0.77 × 10^−2^	0.52 × 10^3^
FAV@B_3_O_3_-SW1						
66	O56-H1	0.54 × 10^−2^	0.19 × 10^−1^	0.38 × 10^−2^	−0.29 × 10^−2^	0.93 × 10^−3^
109	N49-O40	0.79 × 10^−2^	0.26 × 10^−1^	0.59 × 10^−2^	−0.53 × 10^−2^	0.60 × 10^−3^
119	F51-H5	0.36 × 10^−2^	0.16 × 10^−1^	0.31 × 10^−2^	−0.20 × 10^−2^	0.11 × 10^−2^

**Table 3:** The electronic descriptors include the energy gap (E_L-H_), softness (s), hardness (η), chemical potential (μ) and electrophilicity index (ω) in eV for the FAV@B_3_O_3_-C, FAV@B_3_O_3_-C1, FAV@B_3_O_3_-C and FAV@B_3_O_3_-SW1 complexes.

Complexes	E_HOMO_	E_LUMO_	E_L-H_	s	η	μ	ω
B_3_O_3_	−10.42	−0.46	9.96	0.10	4.98	−5.44	2.96
Favipiravir	−9.51	−0.72	8.79	0.11	4.40	−5.11	2.97
FAV@B_3_O_3_-C	−9.53	−0.89	8.64	0.12	4.32	−5.21	3.14
FAV@B_3_O_3_-C1	−9.54	−0.85	8.69	0.11	4.34	−5.20	3.11
FAV@B_3_O_3_-C3	−9.59	−0.97	8.62	0.12	4.31	−5.28	3.23
FAV@B_3_O_3_-SW1	−9.59	−0.88	8.71	0.11	4.36	−5.23	3.13

**Table 4:** The maximum wavelength (*λ*_max_ in nm), oscillating strength (*(f*_o_) and excitation energy (in eV) of bare B_3_O_3_, favipiravir, FAV@B_3_O_3_-C, FAV@B_3_O_3_-C1, FAV@B_3_O_3_-C3 and FAV@B_3_O_3_-SW1 complexes.

Complex	Wavelength (nm)	Oscillating Strength *(f*_o_)	Excitation energy (eV)
B_3_O_3_	221	0.098	5.62
Favipiravir	265	0.179	4.67
FAV@B_3_O_3_-C	273	0.170	4.54
FAV@B_3_O_3_-C1	266	0.119	4.66
FAV@B_3_O_3_-C3	274	0.154	4.53
FAV@B_3_O_3_-SW1	269	0.182	4.60

## Data Availability

Data will be made available to the corresponding author upon request.
